# Tiotropium in chronic obstructive pulmonary disease – a review of clinical development

**DOI:** 10.1186/s12931-020-01407-y

**Published:** 2020-07-29

**Authors:** Antonio Anzueto, Marc Miravitlles

**Affiliations:** 1 Internal Medicine, Pulmonary Disease, University of Texas Health, and South Texas Veterans Health Care System, San Antonio, TX USA; 2 Pneumology Department, Hospital Universitary Vall d’Hebron/Vall d’Hebron Research Institute (VHIR). CIBER de Enfermedades Respiratorias (CIBERES), Barcelona, Spain

**Keywords:** Chronic obstructive pulmonary disease, Dry-powder inhaler, Dyspnea, Exacerbations, HandiHaler®, Long-acting muscarinic antagonist, Lung function, Respimat®, Soft mist inhaler, Tiotropium

## Abstract

**Background:**

Bronchodilators are the mainstay of pharmacological treatment in chronic obstructive pulmonary disease (COPD), and long-acting muscarinic antagonist (LAMA) monotherapy is recommended as initial treatment for Global Initiative for Chronic Obstructive Lung Disease (GOLD) groups B, C, and D.

**Main body:**

Tiotropium bromide was the first LAMA available for COPD in clinical practice and, because of its long duration of action, is administered once daily. Tiotropium was initially available as an inhalation powder delivered via a dry-powder inhaler (DPI). Later, tiotropium also became available as an inhalation spray delivered via a soft mist inhaler (SMI). The SMI was designed to overcome or minimize some of the issues associated with other inhaler types (eg, the need for strong inspiratory airflow with DPIs). Results of short- and long-term randomized, controlled clinical trials of tiotropium in patients with COPD indicated tiotropium was safe and significantly improved lung function, health-related quality of life, and exercise endurance, and reduced dyspnea, lung hyperinflation, exacerbations, and use of rescue medication compared with placebo or active comparators. These positive efficacy findings triggered the evaluation of tiotropium in fixed-dose combination with olodaterol (a long-acting β_2_-agonist). In this review, we provide an overview of studies of tiotropium for the treatment of COPD, with a focus on pivotal studies.

**Conclusion:**

Tiotropium is safe and efficacious as a long-term, once-daily LAMA for the maintenance treatment of COPD and for reducing COPD exacerbations. The SMI generates a low-velocity, long-duration aerosol spray with a high fine-particle fraction, which results in marked lung drug deposition. In addition, high inspiratory flow rates are not required.

## Background

Bronchodilators are the mainstay of pharmacological treatment in chronic obstructive pulmonary disease (COPD) [1]. The 2020 update of the Global Initiative for Chronic Obstructive Lung Disease (GOLD) strategy document includes a model for initiation of pharmacological treatment, which is based on assessment of symptoms and exacerbation risk according to the ABCD assessment tool [2]. After initial treatment, the GOLD committee recommends regular review of symptoms; assessment of inhaler technique and adherence, as well as non-pharmacological approaches; and treatment adjustment as needed. A separate follow-up treatment algorithm is provided that is based on the predominant treatable trait (dyspnea or exacerbations) and current treatment, and is independent of ABCD group at initiation of treatment [2]. Long-acting muscarinic antagonist (LAMA) monotherapy is recommended as initial treatment for GOLD groups B, C, and D [2]. Alternatively, long-acting β_2_-agonist (LABA) monotherapy can be used as initial treatment for GOLD group B, and dual bronchodilator therapy with a LABA/LAMA combination may be considered for group B patients with severe breathlessness. Dual bronchodilator therapy also is an initial treatment option for group D patients who are highly symptomatic (COPD Assessment Test [CAT] score > 20), and a combination of a LABA and inhaled corticosteroid (ICS) is an initial treatment option for group D patients with a blood eosinophil count of 300 cells/μL or more. In general, ICS-based therapy recommendations are guided by exacerbation history, blood eosinophil counts, and coexistent asthma in the GOLD 2020 update [2].

LAMAs inhibit the bronchoconstrictor effect of acetylcholine by prolonged binding to the M3 muscarinic receptors present on airway smooth muscles and faster dissociation from M2 receptors [2, 3]. Tiotropium bromide, the first LAMA available for COPD in clinical practice, with a dissociation half-life of 35 h from the M3 receptor, is structurally related to ipratropium [4, 5]. Ipratropium has a short duration of action, with a dissociation half-life of 0.3 h from the M3 receptor and requires four-times-a-day (QID) dosing, potentially affecting adherence to therapy [4, 5]. In contrast, tiotropium has a long duration of action, enabling once-daily (QD) dosing [5]. Tiotropium was first available as an inhalation powder delivered via a dry-powder inhaler (DPI; Spiriva® HandiHaler®; Boehringer Ingelheim Pharmaceuticals, Inc) and later became available as an inhalation spray delivered via a soft mist inhaler (SMI; Spiriva® Respimat®; Boehringer Ingelheim Pharmaceuticals, Inc). Tiotropium HandiHaler® and tiotropium Respimat® are indicated for long-term, QD maintenance treatment of bronchospasm associated with COPD and for reducing COPD exacerbations in the United States (US), European Union (EU), and other countries [6, 7]. A LABA/LAMA combination is used to leverage their different mechanisms of action. While LAMAs prevent bronchoconstriction, LABAs relax bronchial smooth muscle and cause bronchodilation by stimulating β2-adrenergic receptors in airway smooth muscle which trigger cellular pathways [8]. Tiotropium in a fixed-dose combination with the LABA olodaterol (Stiolto® Respimat® inhalation spray [Spiolto® Respimat® inhalation solution in Europe]; Boehringer Ingelheim Pharmaceuticals, Inc) is indicated for the long-term, QD maintenance treatment of patients with COPD [9, 10].

DPIs, pressurized metered-dose inhalers (pMDIs), and SMIs are used for delivery of inhaled COPD medications. However, patients with COPD might not be able to generate the inspiratory flow necessary for optimal drug de-aggregation and lung deposition with DPIs [11]. DPIs with varying internal resistances are available [11], and patients’ peak inspiratory flow should ideally be assessed before prescribing a DPI [12]. Further, the velocity of the aerosol from pMDIs and the inability of some patients with COPD to properly coordinate actuation and inhalation when using pMDIs can lead to considerable oropharyngeal drug deposition [11]. Use of a spacer or holding chamber with a pMDI helps slow the velocity of aerosolized particles; removes larger, nonrespirable particles; and reduces oropharyngeal deposition [13]. Respimat®, the only available SMI, is a propellant-free inhaler that uses mechanical energy to generate a fine, slow-moving mist [14]. Respimat® provides a higher lung drug deposition than pMDIs or DPIs [15–17], delivers the dose of medication independent of the patient’s inspiratory effort [18], and requires minimal coordinated actuation and inhalation [19, 20], thereby making it suitable for a range of patients with COPD [14].

**Fig. 1:**
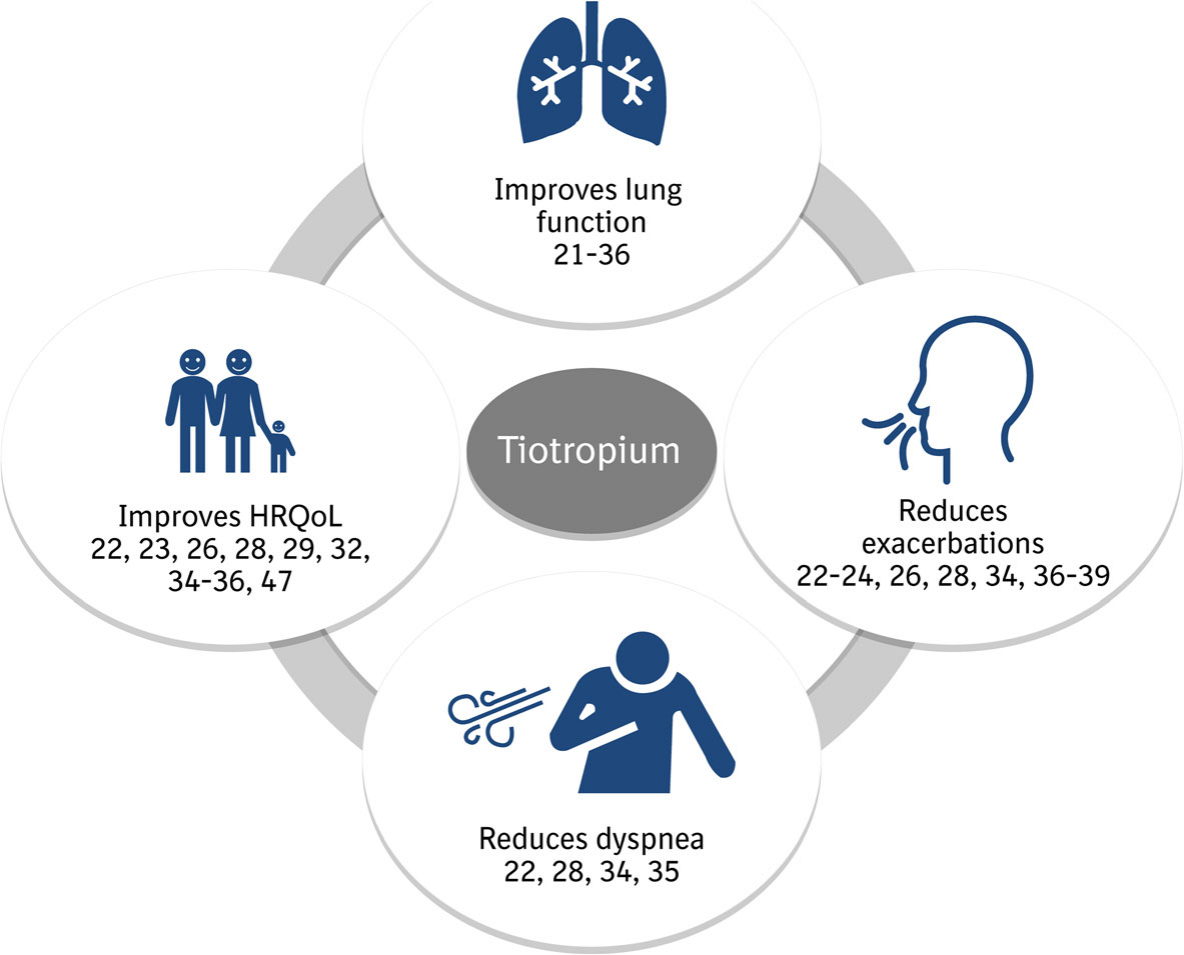
Effects of tiotropium in COPD. *COPD* chronic obstructive pulmonary disease, *HRQoL* health-related quality of life

Cumulative results of clinical trials indicate that tiotropium significantly improves lung function (vs placebo [21–33], ipratropium [27, 34], or salmeterol [35, 36]), reduces dyspnea (vs placebo [22, 28], salmeterol [35], or ipratropium [34]), reduces exacerbations (vs placebo [22–24, 26, 28, 36–38], ipratropium [34], or salmeterol [39]), and improves health-related quality of life (HRQoL; vs placebo [22, 23, 26, 28, 29, 32, 35, 36] or ipratropium [34]; Fig. 1). As reviewed here, most of the evidence base for use of tiotropium in COPD was derived from studies of the initial dry powder formulation. Results of studies of tiotropium as an inhalation solution delivered via an SMI confirmed its long-term efficacy benefits and safety profile. The clinical development of tiotropium in COPD culminated with evidence supporting its use in fixed-dose combination with olodaterol. The objective of this review is to comprehensively detail the key clinical trial evidence for use of tiotropium monotherapy in COPD.

## Initial studies with tiotropium inhalation powder

### Dose response and comparison with ipratropium

In a 4-week, dose-response study of tiotropium inhalation powder (4.5, 9, 18, and 36 μg QD) in patients with COPD (n = 169), all doses significantly improved lung function compared with placebo (day 29; difference in trough forced expiratory volume in 1 s [FEV_1_] response = 0.14 L, 0.11 L, 0.15 L, and 0.19 L, respectively; *p* < 0.05 for all) [31]. Similarly, forced vital capacity (FVC) was higher for all doses of tiotropium than with placebo (day 29; difference in trough FVC response = 0.31 L, 0.22 L, 0.37 L, and 0.22 L, respectively; *p* < 0.05 for 4.5-μg and 18.0-μg doses). All tiotropium doses also increased peak expiratory flow rate (PEFR) compared with placebo at all test points (morning, noon, and evening) [31]. Improvement in lung function was maintained throughout the treatment period. The overall safety profile of the four tiotropium doses was similar to that of placebo [31]. Based on these results, a dosage of 18 μg QD was evaluated in a 3-month, randomized controlled trial (RCT; n = 470) [21]. Again, tiotropium significantly improved lung function compared with placebo (day 92; difference in trough FEV_1_ response = 0.15 L; difference in trough FVC response = 0.28 L; *p* < 0.001 for both), and improvements were maintained throughout the treatment period. Tiotropium also significantly improved daily morning and evening PEFR and reduced symptoms of wheezing, shortness of breath, and “as-needed” albuterol use. Safety of tiotropium was similar to that of placebo, except for dry mouth, which was more common with tiotropium than with placebo (9.3% vs 1.6%; *p* < 0.05) [21]. Further, the efficacy and safety of tiotropium 18 μg QD was compared with that of ipratropium 40 μg QID (via a pMDI) in a 13-week study (n = 288) [40]. Tiotropium improved lung function compared with ipratropium throughout the treatment period (day 92; mean difference in trough FEV_1_ response = 0.13 L [*p* = 0.0001]; mean difference in trough FVC response = 0.21 L [*p* = 0.0003]). Tiotropium also consistently improved weekly mean morning and evening PEFR and reduced the use of rescue salbutamol. As observed in other studies, dry mouth was more common with tiotropium than with ipratropium (14.7% vs 10.3%) [40].

### Pivotal trials

In a 6-month RCT (n = 623), tiotropium 18 μg QD compared with the LABA salmeterol 50 μg twice daily (BID; delivered via a pMDI) significantly improved lung function (week 24; difference in trough FEV_1_ response = 0.052 L; difference in trough FVC response = 0.112 L; *p* < 0.01 for both) and reduced dyspnea (week 24; difference in transition dyspnea index [TDI] focal score = 0.78 U; *p* < 0.05) [35]. Both active drugs significantly reduced the need for rescue albuterol compared with placebo (*p* < 0.0001). In this study, tiotropium also significantly improved HRQoL compared with placebo (week 24; difference in St. George’s Respiratory Questionnaire [SGRQ] total score = − 2.71 U; *p* < 0.05) but not salmeterol (difference = − 1.6 U; *p* > 0.05). Safety of tiotropium was similar to that of placebo and salmeterol, except for dry mouth, which was more common with tiotropium (10%) [35]. Health outcomes were evaluated in two 6-month RCTs (n = 1207) in which tiotropium 18 μg compared with placebo significantly reduced the number of exacerbations/patient-year (PY; 1.07 vs 1.49; *p* < 0.05), increased the time to first exacerbation (*p* ≤ 0.01), and improved HRQoL (difference in SGRQ total score = − 2.7 U; *p* < 0.01) during the treatment period [36]. Salmeterol did not significantly differ from placebo for these outcomes. Although both active drugs improved lung function, tiotropium did so to a greater extent than salmeterol. Dry mouth was more common with tiotropium (8.2%) than with salmeterol (1.7%) or placebo (2.3%) [36].

In two identical 12-month RCTs (n = 921), tiotropium 18 μg QD significantly improved lung function compared with placebo (difference in trough FEV_1_ response = 0.12–0.15 L [range over days 1–344]; *p* < 0.01) [22]. In addition, compared with patients who received placebo, those who received tiotropium also had significantly less dyspnea (difference in TDI focal score = 0.8–1.1 U [range over days 50–344]; *p* < 0.001), better health status scores (*p* < 0.05), and fewer COPD exacerbations/PY (0.76 vs 0.95; *p* = 0.045) and exacerbation-related hospitalizations (*p* < 0.05). In this and other tiotropium trials, patients were allowed to continue taking glucocorticoids during the study period. As observed in prior studies, incidence of dry mouth was higher with tiotropium than with placebo (16.0% vs 2.7%; *p* < 0.05). In two other identical 12-month RCTs (n = 535), tiotropium 18 μg QD compared with ipratropium 40 μg QID (delivered via a pMDI) significantly improved lung function (difference in trough FEV_1_ response = 0.15 L; *p* < 0.001) and reduced the use of rescue salbutamol (*p* < 0.05) at the end of the treatment period [34]. In addition, tiotropium compared with ipratropium significantly reduced dyspnea (day 364; difference in TDI focal score = 0.90 U; *p* = 0.001) and the number of exacerbations/PY (0.73 vs 0.96; *p* = 0.006); improved PEFR (difference in morning PEFR = 10–18 L/min [range over days 1–365]; difference in evening PEFR = 9–18 L/min [range over days 1–365]; *p* < 0.01) and HRQoL (day 364; difference in SGRQ total score = − 3.30; *p* = 0.004); and increased the time to first exacerbation (*p* = 0.008) and time to first exacerbation-related hospitalization (*p* = 0.048). These improvements were maintained throughout the treatment period. Collectively, results of these studies showed that QD tiotropium 18 μg was a safe and efficacious LAMA for maintenance treatment of COPD and for reducing exacerbations, leading to the approval of Spiriva® HandiHaler® in the EU (2002), US (2004), and other countries [41, 42].

### Effect on lung hyperinflation, exercise endurance, exertional dyspnea, and HRQoL

Patients with COPD often experience hyperinflation, which results in reduced inspiratory capacity (IC), limited exercise capacity, and increased exertional dyspnea [2, 43]. A few RCTs were designed to specifically evaluate the effects of tiotropium on these parameters.

In a 4-week RCT (n = 81), tiotropium 18 μg QD significantly improved lung function and IC compared with placebo (mean differences at week 4: trough FEV_1_ = 0.16 L; trough FVC = 0.33 L; trough IC = 0.22 L; *p* < 0.01 for all) [44]. Similarly, in a 6-week RCT (n = 187), tiotropium significantly improved lung function compared with placebo, consistent with findings from prior studies [45]. Further, tiotropium compared with placebo significantly reduced lung hyperinflation (residual volume, *p* < 0.001; functional residual capacity, *p* < 0.001); increased vital capacity (*p* < 0.0001), IC (difference in trough response = 0.10 L; *p* < 0.05), and exercise endurance (difference in endurance time = 105 s [21%]; *p* < 0.01); and decreased exertional dyspnea (*p* < 0.01) on day 42 [45]. Change from baseline in all parameters was evident after the first treatment and persisted for the duration of the trial. In a subsequent 6-week study (n = 261), effects of tiotropium on lung hyperinflation, symptom-limited exercise tolerance, and exertional dyspnea were apparent at 2.25 h of treatment and lasted for 8 h after dosing on day 42 [46]. These findings were corroborated by results of another RCT (n = 100), where tiotropium 18 μg compared with placebo not only significantly improved trough FVC (difference = 0.20 L; *p* < 0.05) and trough IC (difference = 0.15 L; *p* < 0.05) but also significantly increased mean distance walked during the shuttle-walk test (difference = 36 m; *p* < 0.05) and improved HRQoL (difference in SGRQ total score = − 6.5; *p* = 0.026) after 12 weeks of treatment [32]. Further, in an RCT (n = 554) by the Tiotropium: Influence sur la Perception de l’amélioration des activites Habituelles Objectivée par une echelle Numerique (TIPHON) group—in addition to significantly improving lung function (difference in trough FEV_1_ = 0.10 L; *p* = 0.0001) and reducing exacerbations/PY (1.05 vs 1.83; *p* = 0.0287)—tiotropium compared with placebo significantly increased the proportion of patients achieving clinically relevant improvement in HRQoL (difference in responders = 10.9%; *p* = 0.029) after 9 months of treatment [23]. In contrast to the results of the above studies, in a 96-week RCT (n = 519), the difference in endurance time between tiotropium 18 μg QD and placebo was not statistically significant (tiotropium/placebo = 1.13; 95% CI = 0.97–1.32; *p* = 0.106). However, consistent with previous studies, tiotropium improved HRQoL at 96 weeks compared with placebo (difference in SGRQ total score = 4.03 U; *p* = 0.007) [47].

### Effect on exacerbations

Patients with COPD, even those with mild disease, can experience exacerbations [48–51], which account for a large proportion of total COPD burden on the healthcare system [52, 53]. Recommendations for reducing COPD exacerbations and treatment of stable COPD have been provided by GOLD [2], the European Respiratory Society/American Thoracic Society [54], and the Spanish Society of Pulmonology and Thoracic Surgery [55, 56]. The efficacy of tiotropium 18 μg QD in reducing exacerbations was evaluated in several short- and long-term studies, including those mentioned above (Table 1**;** summarized by Anzueto et al. [57]).

**Table 1:** Tiotropium reduces exacerbation and increases time to first exacerbation

Study	Comparator	Patients (N)	Study duration	Change from baseline in number of exacerbations	Change from baseline in patients with ≥ 1 exacerbation	Increase in time to first exacerbation
Casaburi 2002 [22]	Placebo	921	1 year	−20% (*p* = 0.045)	− 14% (*p* < 0.05)	*p* = 0.011
Vincken 2002 [34]	Ipratropium	535	1 year	−24% (*p* = 0.006)	−24% (*p* = 0.014)	*p* = 0.008
Brusasco 2003 [36]	Placebo	802^a^	6 months	−28% (*p* < 0.05)	−18% (*p* > 0.05)	*p* ≤ 0.01
Niewoehner 2005 [37]	Placebo	1829	6 months	−19% (*p* = 0.031)	−14% (*p* = 0.037)	*p* = 0.028
Dusser 2006 [24]	Placebo	1010	1 year	−35% (*p* < 0.001)	− 17% (*p* < 0.01)	*p* < 0.001
Tashkin 2008 [26]	Placebo	5993	4 years	− 14% (*p* < 0.001)	−2% (*p* = 0.35)	*p* < 0.001
Tonnel 2008 [23]	Placebo	554	9 months	−43% (*p* = 0.0287)	− 16% (*p* = 0.1013)	*p* = 0.0081
Bateman 2010 [28]	Placebo	1990	1 year	*p* < 0.01**	−16% (*p* < 0.01)^b^	*p* < 0.0001^b^
Bateman 2010 [29]	Placebo	3991	48 weeks	−21% (*p* < 0.0001)	− 18% (*p* < 0.0001)	*p* < 0.0001
Vogelmeier 2011 [39]	Salmeterol	7376	1 year	−11% (*p* = 0.002)^c^	−11% (*p* < 0.001)^c^	*p* < 0.001

**p < 0.01 for tiotropium 5 μg dose and *p* < 0.001 for tiotropium 10 μg dose

^a^This number does not include the patients in the salmeterol group. ^b^For both tiotropium 5-μg and 10-μg doses. ^c^Refers to moderate or severe exacerbations

In an RCT (n = 1829) conducted in a US Veterans Affairs setting, fewer patients treated with tiotropium than placebo experienced ≥1 exacerbation (difference = − 5.7%; *p* = 0.037) or exacerbation-related hospitalization (difference = − 3.0%; *p* = 0.056) after 6 months of treatment [37]. Analysis of secondary outcomes indicated that tiotropium significantly increased time to first exacerbation (*p* = 0.028) and reduced health care resource utilization (HCRU; frequency of hospitalizations, *p* = 0.047; antibiotic treatment days, *p* = 0.015; and unscheduled clinic visits, *p* = 0.019). Similar results were observed in another RCT (n = 1010; Mesure de l’Influence de Spiriva® sur les Troubles Respiratoires Aigus à Long terme [MISTRAL]) [24], where tiotropium compared with placebo significantly increased time to first exacerbation (*p* < 0.001) and reduced the number of exacerbations/PY (1.57 vs 2.41; *p* < 0.001), proportion of patients with ≥1 exacerbation (difference = − 10.4%; *p* < 0.01), and HCRU (concomitant respiratory medications, *p* < 0.0001; antibiotics, *p* < 0.001; and oral steroids, *p* < 0.01; and the number of unscheduled physician contacts, *p* < 0.05) after treatment for 1 year [24]. Tiotropium also significantly improved weekly morning PEFR (mean difference over 1 year = 25 L/min; *p* < 0.0001), trough FEV_1_ (mean difference = 0.12 L; *p* < 0.0001), FVC (mean difference = 0.17 L; *p* < 0.0001), and IC (mean difference = 0.14 L; *p* < 0.001) at the end of the treatment period. As observed in other studies, the safety of tiotropium was similar to that of placebo, except for dry mouth, which was more frequent with tiotropium (4.0%) than with placebo (1.4%) [24]. In addition, in the Prevention Of Exacerbations with Tiotropium in COPD (POET-COPD) trial (n = 7376), tiotropium 18 μg QD was significantly more efficacious than salmeterol 50 μg BID in increasing the time to first exacerbation (hazard ratio [HR] = 0.83 [ie, 17% reduction in risk of exacerbations with tiotropium]; *p* < 0.001) and reducing the annual rate of moderate or severe exacerbations (0.64 vs 0.72; rate ratio [RR] = 0.89 [11% reduction with tiotropium]; *p* = 0.002) and severe exacerbations (0.09 vs 0.13; RR = 0.73 [ie, 27% reduction with tiotropium]; *p* < 0.001) after 1 year of treatment [39]. The safety of tiotropium was similar to that of salmeterol. In a post hoc analysis of POET-COPD, tiotropium compared with salmeterol increased the time to first exacerbation and reduced the number of exacerbations in patients at low and high risk of exacerbation (time to first exacerbation: HR = 0.89; *p* = 0.1046 and HR = 0.84; *p* = 0.0002, respectively; number of exacerbations: RR = 0.89; *p* = 0.1768 and RR = 0.90; *p* = 0.0383, respectively) [58].

### Longer-term studies

Longer-term studies were designed to expand upon findings from the 6- and 12-month RCTs. In a 2-year RCT (n = 841) of patients with early-stage COPD (ie, GOLD stage 1 [mild] or 2 [moderate]) in China, tiotropium HandiHaler® 18 μg QD compared with placebo significantly improved lung function throughout the 2-year period (range of mean differences in FEV_1_: before bronchodilator use = 0.127–0.169 L; after bronchodilator use = 0.071–0.133 L; *p* < 0.001 for both) [25]. In addition, in the Understanding Potential Long-Term Impacts on Function with Tiotropium (UPLIFT) trial (n = 5993), tiotropium compared with placebo significantly improved lung function (range of mean differences in FEV_1_: before bronchodilator use = 0.087–0.103 L; after bronchodilator use = 0.047–0.065 L; *p* < 0.001 for both), reduced the number of exacerbations/PY (0.73 vs 0.85; relative risk = 0.86 [ie, 14% reduction with tiotropium]; *p* < 0.001), increased the time to first exacerbation (*p* < 0.001), improved HRQoL (mean difference in SGRQ total score = − 2.7 U; *p* < 0.001), and reduced mortality (HR = 0.87; 95% confidence interval [CI] = 0.76–0.99) in patients with moderate-to-very severe COPD treated for 4 years [26]. Tiotropium significantly improved FEV_1_ and HRQoL compared with placebo throughout the trial.

Conflicting results were reported, however, with respect to the effect of tiotropium on annual decline in FEV_1_. Tiotropium compared with placebo significantly reduced the annual decline in FEV_1_ after bronchodilator use in the aforementioned 2-year RCT in China [25] (mean decline in FEV_1_ from day 30 to month 24: before bronchodilator use = 0.038 L/year vs 0.053 L/year [*p* = 0.06]; after bronchodilator use = 0.029 L/year vs 0.051 L/year [*p* = 0.006]) and in a retrospective analysis of two 1-year RCTs (mean decline in trough FEV_1_ from days 8 to 344 = 0.012 vs 0.058 L/year; *p* = 0.005) [59]. In UPLIFT, however, the annual decline in FEV_1_ was not significantly different between the tiotropium and placebo groups (mean decline in FEV_1_ from day 30 to month 48: before bronchodilator use = 0.030 L/year vs 0.030 L/year [*p* = 0.95]; after bronchodilator use = 0.040 L/year vs 0.042 L/year [*p* = 0.21]) [26]. In a pre-specified subgroup analysis of the UPLIFT trial in patients with GOLD stage II COPD, the annual decline in post-bronchodilator FEV_1_ was lower in the tiotropium group compared with the placebo group (mean decline in post-bronchodilator FEV_1_ from day 30 to month 48: 0.043 L/year vs 0.049 L/year [p = 0.024]), supporting benefits of early intervention in COPD [60].

Long-term effects of tiotropium as first-line maintenance medication in COPD were further evaluated in a secondary analysis of data from the UPLIFT trial [61]. In addition, various post hoc and subgroup analyses were conducted to assess the long-term efficacy of 4 years of treatment with tiotropium with regard to smoking status [62], sex [63], and baseline FEV_1_ ≥ 60% predicted [64]. In the latter analysis (n = 1210), tiotropium compared with placebo significantly improved lung function (difference in pre-bronchodilator FEV_1_ = 0.087–0.127 L [range over months 1–48]; difference in post-bronchodilator FEV_1_ = 0.038–0.084 L [range over months 1–48]; *p* ≤ 0.002 for both) and HRQoL (difference in SGRQ total score = − 2.0 to − 3.4 U; *p <* 0.05) and reduced the risk of exacerbations (HR = 0.83; 95% CI = 0.71–0.96) and mortality (HR = 0.66; 95% CI = 0.45–0.96) over the 4-year treatment period [64]. In another analysis of the UPLIFT data, patients taking ICS had significantly higher incidence rates of pneumonia (0.068 vs 0.056; *p* = 0.012) and COPD exacerbations (0.88 vs 0.62; *p* < 0.001) than patients not taking ICS [65]. An attenuated rate of pneumonia was observed in the tiotropium subgroup, in general, irrespective of ICS use (pneumonia incidence rate: fluticasone/placebo = 0.081; fluticasone/tiotropium = 0.073; other ICS/placebo = 0.062; other ICS/tiotropium = 0.055; no ICS/placebo = 0.055; and no ICS/tiotropium = 0.056), suggesting the adverse effects associated with the use of ICS might be mitigated with add-on tiotropium.

### Efficacy and safety vs other therapies

#### Tiotropium vs ICS/LABA

Tiotropium 18 μg QD provided a bronchodilatory effect comparable to fluticasone/salmeterol 250/50 μg BID in a 6-week RCT (n = 107) of patients with moderate-to-very severe COPD [66]. In addition, in the Investigating New Standards for Prophylaxis in Reducing Exacerbations (INSPIRE) trial (n = 1323), fluticasone/salmeterol 500/50 μg BID was comparable to tiotropium 18 μg QD in reducing exacerbations (modeled annual exacerbation rate = 1.28 vs 1.32; *p* = 0.656) in patients with severe and very severe COPD after 2 years of treatment [67]. However, fluticasone/salmeterol was associated with an increased incidence of pneumonia compared with tiotropium (*p* = 0.008) [67].

#### Tiotropium vs short-acting muscarinic antagonist/short-acting β_2_-agonist (SAMA/SABA) combinations

Patients with moderate-to-very severe COPD taking ipratropium/albuterol 36/206 μg QID delivered via a pMDI who switched to tiotropium 18 μg QD had significantly improved lung function compared with those who continued taking ipratropium/albuterol (difference in mean trough FEV_1_ at 84 days = 0.086 L; *p* < 0.0001) in a randomized, parallel-group, double-blind, double-dummy study (n = 676) [68]. The incidence of respiratory adverse events was lower in the tiotropium group than in the ipratropium/albuterol group [68]. Additionally, tiotropium significantly reduced the risk of exacerbations (*p* = 0.0086) and COPD-related referrals/hospitalizations (*p* = 0.004) compared with ipratropium/salbutamol at the end of treatment in a retrospective, 12-month, follow-up study (n = 4193) using the United Kingdom General Practice Research Database [69].

#### Tiotropium vs QD LABA

The efficacy of QD indacaterol was compared with that of QD tiotropium in several trials. Indacaterol 150 μg QD and indacaterol 300 μg QD delivered via a DPI (Onbrez® Breezhaler®; Novartis Pharmaceuticals) were at least as effective as tiotropium 18 μg QD in improving lung function and clinical outcomes in patients with moderate-to-severe COPD in short-term trials: INdacaterol & TIotropium: Measuring Efficacy (INTIME; indacaterol 150 or 300 μg; 14 days, n = 169) [70], INdacaterol Towards Establishment of cliNical SuperiorITY study (INTENSITY; indacaterol 150 μg; 12 weeks, n = 1598) [71], and Indacaterol to Help Achieve New COPD treatment Excellence (INHANCE; indacaterol 150 or 300 μg; 26 weeks, n = 1683) [72]. Further, indacaterol 150 μg QD was non-inferior to tiotropium 18 μg QD in improving lung function (week 12; least squares mean difference in trough FEV_1_ = − 0.011 L; *p* < 0.0001) in patients with severe COPD and a history of ≥1 exacerbation in the previous year in the 52-week Indacaterol: Providing Opportunity to Re-engage Patients with Life (INVIGORATE) trial (n = 3444) [73]. Tiotropium was superior to indacaterol in reducing exacerbations (week 52; annual rate = 0.73 vs 0.90; *p* < 0.001). Safety was similar between the treatment groups.

#### Tiotropium vs other LAMAs

In a 6-week RCT (n = 414), the efficacy of aclidinium 400 μg BID delivered via a DPI (Genuair®/Pressair™; AstraZeneca) was compared with that of placebo and tiotropium 18 μg QD in patients with moderate-to-severe COPD [74]. Both aclidinium and tiotropium compared with placebo significantly improved lung function (differences in change from baseline in FEV_1_ area under the curve for the 24-h period post-morning dose [AUC_0–24_] = 0.15 L and 0.14 L, respectively; both *p* < 0.0001) and COPD symptom scores (*p* < 0.0001 and *p* < 0.05, respectively) at the end of the treatment period. Aclidinium, but not tiotropium, reduced the severity of nighttime symptoms vs placebo (*p* < 0.05). Safety was similar between treatment groups. At week 12 of the glycopyrronium bromide in COPD airways trial 5 (GLOW 5; n = 657), lung function was comparable between glycopyrronium 50 μg QD (Breezhaler®) and tiotropium HandiHaler® 18 μg QD (week 12; least squares mean trough FEV_1_ = 1.405 L for both) [75]. Improvements in dyspnea, HRQoL, rescue medication use, and rate of COPD exacerbations, as well as safety, were also similar between groups.

## Clinical development of tiotropium Respimat®

pMDIs generate aerosols with high velocity, which leads to considerable oropharyngeal drug deposition, and the necessary coordination of inhalation and actuation is difficult for some patients [76]. On the other hand, most DPIs require a strong inspiratory airflow, which also may be difficult for some patients [76].

### Soft mist inhaler

The only available SMI, Respimat®, is a pocket-sized, propellant-free device that generates an aerosol from a drug solution effectively and consistently [76]. Respimat®, which was developed to overcome or minimize some of the limitations of other inhalers [77], uses mechanical energy to generate a fine, slow-moving aerosol cloud (mist) from the drug solution, requires minimal coordination of inhalation and actuation, and does not require high inspiratory flow rates [14]. Further, the fine-particle fraction (approximately 75%; particles > 1 to < 5.8 μm) of the mist is nearly twice that in the aerosol cloud emitted from most pMDIs and DPIs [76], and the mist has a lower velocity and a longer duration than the aerosol cloud of pMDIs (mean velocity at a 10-cm distance from the nozzle: SMI = 0.8 m/s; pMDIs = 2.0–8.4 m/s; mean duration: SMI = 1.5 s; pMDIs = 0.15–0.36 s) [20]. These factors contribute to reduced oropharyngeal drug deposition and increased drug deposition in the lungs [15–17].

### Dose response

Because Respimat® provides high drug lung deposition, lower doses of tiotropium were expected to provide comparable efficacy as the doses used with HandiHaler® [30]. A 3-week, dose-ranging RCT (n = 202) was conducted to compare various tiotropium doses (1.25 μg, 2.5 μg, 5 μg, 10 μg, or 20 μg QD) delivered via Respimat®, placebo delivered via Respimat®, tiotropium 18 μg delivered via HandiHaler®, and placebo delivered via HandiHaler® [30]. Tiotropium Respimat® 5 μg and 20 μg significantly improved lung function compared with placebo Respimat® (difference in trough FEV_1_ response = 0.17 L [both doses]; *p* < 0.05 for both) at the end of treatment. Tiotropium Respimat® 10 μg also improved lung function but not significantly (*p* = 0.06) more than placebo [30]. Safety was similar across treatment groups.

### Pivotal studies

In a pre-specified pooled analysis of two 30-week studies (n = 207), tiotropium Respimat® 5 μg and 10 μg QD were superior to placebo in improving lung function (day 29; difference in trough FEV_1_ response = 0.126 L and 0.127 L, respectively; *p* < 0.0001 for both) and were non-inferior to tiotropium HandiHaler® 18 μg QD (day 29; difference in trough FEV_1_ response = 0.029 L and 0.031 L, respectively; *p* < 0.0001 for both) [78]. In two identical, short-term, randomized, active- and placebo-controlled trials (n = 719), the efficacy and safety of tiotropium Respimat® 5 μg and 10 μg QD were compared with that of ipratropium 36 μg QID delivered via a pMDI [27]. At week 12, both tiotropium doses significantly improved lung function compared with ipratropium (difference in trough FEV_1_ response = 0.064 L [*p* = 0.006] and 0.095 L [*p* < 0.001], respectively; difference in trough FVC response = 0.077 L [*p* > 0.01] and 0.125 L [*p* < 0.01], respectively) and compared with placebo (difference in trough FEV_1_ response = 0.118 L and 0.149 L, respectively [both *p* < 0.0001]; difference in trough FVC response = 0.132 L [*p* < 0.01] and 0.180 L [*p* < 0.0001], respectively) [27]. Both tiotropium doses also significantly reduced rescue medication use compared with placebo (*p* = 0.0061 and *p* < 0.0001, respectively); however, only the 10-μg dose was statistically superior to ipratropium (*p* = 0.04). The long-term efficacy and safety of tiotropium Respimat® 5 μg and 10 μg QD were evaluated in two identical RCTs (n = 1990) [28]. At 1 year of treatment, both tiotropium doses compared with placebo significantly improved lung function (difference in trough FEV_1_ response = 0.127 L and 0.150 L, respectively; both *p* < 0.0001) and HRQoL (difference in SGRQ total score = − 3.5 and – 3.8, respectively; both *p* < 0.0001) and reduced dyspnea (difference in TDI focal score = 1.05 and 1.08, respectively; both *p* < 0.0001) and mean COPD exacerbation rate/PY (odds ratio [OR] = 0.75 [*p* < 0.01] and 0.74 [*p* < 0.001], respectively). Significantly fewer patients experienced ≥1 exacerbation in the tiotropium groups than in the placebo group (difference = − 6.9 and − 7.2%, respectively; both *p* < 0.01). The incidence of gastrointestinal disorders was greater in the tiotropium groups than in the placebo group (dry mouth: 5 μg = 7.2%, 10 μg = 14.5%, placebo = 2.1%; constipation: 5 μg = 2.1%, 10 μg = 2.2%, placebo = 1.5%). In another 1-year RCT (n = 3991), the efficacy and safety of tiotropium Respimat® 5 μg QD were evaluated [29]. Patients were permitted to use usual therapy (any concurrent COPD medications except inhaled anticholinergics) to reflect closely the place of tiotropium in COPD management. At week 48, tiotropium 5 μg compared with placebo significantly improved lung function (adjusted mean difference in trough FEV_1_ = 0.102 L; adjusted mean difference in trough FVC = 0.168 L; both *p* < 0.0001) and improved HRQoL (adjusted mean difference in SGRQ total score = − 2.9 U; *p* < 0.0001). Further, the proportion of patients having ≥1 exacerbation was lower in the tiotropium group compared with the placebo group (35.3 and 43.1%, respectively; HR = 0.69 [ie, 31% reduction in the risk of exacerbations with tiotropium; *p* < 0.0001) [29]. Collectively, results of these studies showed that tiotropium Respimat® 5 μg QD was efficacious as maintenance treatment for COPD and for reducing exacerbations, leading to the approval of Spiriva® Respimat® in the EU (2007), US (2014), and other countries [79].

### Observational studies

The effectiveness of tiotropium Respimat® 5 μg QD was evaluated in a 6-week open-label observational study (n = 1230) [80]. Tiotropium improved physical function as indicated by a significant increase in the mean Physical Function subdomain (PF-10) score (baseline, 49.0 points; week 6, 62.3 points; difference, 13.4 points [p < 0.001]). Further, the proportion of patients achieving a ≥10-point improvement in PF-10 score was not significantly different between smokers and non-smokers (61.4 and 61.6%, respectively; difference, *p* = 0.93). Adverse events were reported by 4.0% of patients, the most common being respiratory symptoms and dry mouth.

### Landmark safety trial

The TIOtropium Safety and Performance In Respimat® (TIOSPIR®) trial (n = 17,315) was conducted to confirm the safety and efficacy of tiotropium Respimat® in a large population of patients with COPD [81]. By having an adequate patient population size (> 17,000 patients at > 1200 investigator sites in 50 countries), broad inclusion criteria to closely reflect real-world patients with COPD, and sufficient treatment duration, analyses of all-cause mortality and time to first COPD exacerbation were possible. During a 2.3-year mean follow-up period, tiotropium Respimat® 5 μg and 2.5 μg QD were non-inferior to tiotropium HandiHaler® 18 μg QD in the risk of death (both *p* < 0.05) and not superior in the risk of exacerbation (*p* = 0.42 and *p* = 0.56, respectively) [81]. In patients with a history of cardiac arrhythmia, tiotropium Respimat® 2.5 μg and tiotropium HandiHaler®18 μg had a similar impact on survival as measured by all-cause mortality. In addition to these analyses, various post hoc analyses were conducted to assess the impact of geographical variations [82] on COPD outcomes, to evaluate spirometry outcomes [83], and to determine risk factors for exacerbations [84]. Tiotropium Respimat® 5 μg was associated with a similar improvement in trough FVC and a comparable rate of decline in FEV_1_ as tiotropium HandiHaler® 18 μg in the spirometry outcomes analysis [83]. In a multivariate analysis, baseline pulmonary maintenance medication was predictive of, and ICS use was associated with, increased exacerbation risk [84].

TIOSPIR® was conducted because a numerical, but not statistically significant, imbalance in the number of deaths had been noted in meta-analyses of Respimat® trials, between tiotropium Respimat® and placebo Respimat® or tiotropium HandiHaler®, particularly for patients with known cardiac disorders [85–87]. Valid concerns were quickly raised, however, about the meta-analyses’ methodologies and conclusions, particularly the analysis conducted by Singh et al. [85], which included five of the aforementioned RCTs [27–29, 88–90]. For example, in that analysis, conclusions were based on three 1-year studies that had mortality imbalances (and not based on two 12-week studies that showed no imbalance) [88]. Additionally, in one of the studies with mortality imbalances, the placebo arm had an unusually low (0.77%) mortality rate compared with that observed in other studies (1.5–2.5% or higher) [88]. Further, fatal cases were incorrectly assigned to treatment groups [88, 91], and a 6-month trial of > 850 patients [92], which had only two deaths in the tiotropium Respimat® arm and five deaths in the placebo Respimat® arm, was not included [88, 91]. Moreover, two doses of tiotropium—the marketed 5-μg dose and a non-approved, unmarketed 10-μg dose—were included in the primary analysis [91, 93]. These and other factors provide a plausible explanation for the imbalances observed in the aforementioned meta-analyses. In TIOSPIR®, a similar impact on survival was observed between tiotropium Respimat® 2.5 μg and tiotropium HandiHaler® 18 μg, which expands upon findings from UPLIFT and reinforces the safety and efficacy of tiotropium in patients with COPD, regardless of cardiac arrhythmia history [26, 29, 81].

Lastly, results of an analysis of the tiotropium COPD clinical program (Table 2) showed that the characteristics of patients included in the RCTs were representative of “real-life” patient populations, thus demonstrating external validation of the results [94]. Further, tiotropium Respimat® 2.5 μg or 5 μg had similar exacerbation efficacy and safety to that of tiotropium HandiHaler® 18 μg [81].

**Table 2:** Tiotropium – summary table of evidence

Study	Patients (N)	Treatment arms	Primary endpoint results*	Proportion of patients with adverse events	Conclusion of the study
Casaburi 2000 [21]	470	• Tiotropium 18 μg QD • Placebo	Trough FEV_1_ response: 0.11 L vs − 0.04 L (*p* < 0.001)	Overall adverse events: 61.6% vs 66.5% Dry mouth: 9.3% vs 1.6% (*p* < 0.05)	Tiotropium was safe and effective
Casaburi 2002 [22]	921	• Tiotropium 18 μg QD • Placebo	Trough FEV_1_ response: 0.11 L to 0.13 L (tiotropium; *p* < 0.01 vs placebo)	Overall adverse events: 90.0% vs 91.1% Dry mouth: 16.0% vs 2.7% (*p* < 0.05)	Tiotropium significantly improved lung function and HRQoL and reduced dyspnea, COPD exacerbations, and hospitalizations
Vincken 2002 [34]	535	• Tiotropium 18 μg QD • Ipratropium 40 μg QID	Trough FEV_1_ response: 0.12 L vs − 0.03 L (*p* < 0.001)	Adverse events leading to discontinuation: 10.1% vs 12.8% Dry mouth: 12.1% vs 6.1% (*p* = 0.03)	Tiotropium significantly improved lung function and HRQoL and reduced dyspnea and COPD exacerbations compared with ipratropium
Donohue 2002 [35]	623**	• Tiotropium 18 μg QD • Salmeterol 50 μg BID	Trough FEV_1_ response: 0.14 L vs 0.09 L (*p* < 0.01)	Dry mouth: 10% vs NA	Tiotropium significantly improved lung function and reduced dyspnea compared with salmeterol
Brusasco 2003 [36]	1207	• Tiotropium 18 μg QD • Salmeterol 50 μg BID • Placebo	COPD exacerbation rate: 1.07 vs 1.23 vs 1.49 (*p* < 0.05 for tiotropium vs placebo)	Dry mouth: 8.2% vs 1.7% vs 2.3%	Tiotropium significantly improved lung function compared with salmeterol; improved HRQoL and reduced dyspnea and COPD exacerbations compared with placebo
O’Donnell 2004 [45]	187	• Tiotropium 18 μg QD • Placebo	Difference in endurance time between tiotropium and placebo: 105 s (*p* = 0.0098)	Overall adverse events: 36.7% vs 41.0%	Tiotropium significantly reduced lung hyperinflation at rest and exercise and improved exertional dyspnea and endurance time
Niewoehner 2005 [37]	1829	• Tiotropium 18 μg QD • Placebo	Percentage of patients with ≥1 exacerbation: 27.9% vs 32.3% (*p* = 0.037) Percentage of patients with COPD-related hospitalization: 7.0% vs 9.5% (*p* = 0.056)	Serious adverse events: 18% vs 17%	Tiotropium reduced COPD exacerbations, COPD-related hospitalization, and healthcare utilization compared with placebo
Dusser 2006 [24]	1010	• Tiotropium 18 μg QD • Placebo	Percentage of patients with ≥1 exacerbation: 49.9% vs 60.3% (*p* < 0.01)	Overall adverse events: 46.4% vs 45.1% Dry mouth: 4.0% vs 1.4%	Tiotropium significantly improved lung function and reduced COPD exacerbations and COPD-associated health resource use compared with placebo
Verkindre 2006 [32]	100	• Tiotropium 18 μg QD • Placebo	Trough FVC: Difference: 0.20 L (*p* < 0.05)	Adverse events leading to discontinuation: 2% vs 11% Dry mouth: 2% vs 0%	Tiotropium significantly improved FVC, lung hyperinflation, walking distance, and HRQoL
Bateman 2008 [66]	107	• Tiotropium 18 μg QD • Fluticasone/salmeterol 250/50 μg BID	FEV_1_ AUC_0–12h_: 1.55 L vs 1.57 L (*p* = 0.63)	Overall adverse events: 41.1% vs 43.1% Dry mouth: 3.6% vs 3.9%	Tiotropium improved lung function similar to fluticasone/salmeterol combination
Tashkin 2008 [26]	5993	• Tiotropium 18 μg QD • Placebo	Rate of decline in FEV_1_ before bronchodilation: 0.030 L vs 0.030 L (*p* = 0.95) Rate of decline in FEV_1_ after bronchodilation: 0.040 L vs 0.042 L (*p* = 0.21)	Overall adverse events: 92.6% vs 92.3%	Tiotropium significantly improved lung function, improved HRQoL, reduced exacerbations, but did not significantly reduce rate of decline in FEV_1_ compared with placebo
Tonnel 2008 [23]	554	• Tiotropium 18 μg QD • Placebo	Proportion of patients with improvement in HRQoL^a^: 59.1% vs 48.2% (*p* = 0.029)	Patients with ≥1 adverse event: 60.9% vs 67.0% Dry mouth: 1.1% vs 0.7%	Tiotropium significantly improved lung function, improved HRQoL, and reduced exacerbations
Voshaar 2008 [27]	719	• Tiotropium 5 μg QD • Tiotropium 10 μg QD • Ipratropium 36 μg QID vs placebo	Trough FEV_1_ response treatment differences: tiotropium 5 μg − placebo: 0.118 L (*p* < 0.0001) tiotropium 10 μg − placebo: 0.149 L (*p* < 0.0001) tiotropium 5 μg − ipratropium: 0.064 L (*p* = 0.006) tiotropium 10 μg − ipratropium: 0.095 L (*p* < 0.0001)	Overall adverse events: 52.8% vs 60.0% vs 59.6% vs 59.1% Dry mouth: 8.3% vs 10.0% vs 3.9% vs 2.2%	Tiotropium (via Respimat®) significantly improved lung function compared with ipratropium (pMDI) and placebo
Wedzicha 2008 [67]	1323	• Tiotropium 18 μg QD • Fluticasone/salmeterol 500/50 μg BID	Modeled annual rate of exacerbations: 1.32 vs 1.28 (*p* = 0.656)	Overall adverse events: 62% vs 66%	Tiotropium was similar to fluticasone/salmeterol in exacerbation efficacy
Bateman 2010 [28]	1990	• Tiotropium 5 μg QD • Tiotropium 10 μg QD • Placebo	Trough FEV_1_ response: tiotropium 5 μg vs placebo: 0.127 L (*p* < 0.0001) tiotropium 10 μg vs placebo: 0.150 L (*p* < 0.0001)	Overall adverse events: 75.4% vs 78.7% vs 76.9%	Tiotropium (via Respimat®) significantly improved lung function and HRQoL and reduced dyspnea and exacerbations compared with placebo
Bateman 2010 [29]	3991	• Tiotropium 5 μg QD • Placebo	Trough FEV_1_ response: 0.119 L vs 0.018 L (*p* < 0.0001) Time to first exacerbation: 169 days vs 119 days (*p* < 0.0001)	Overall adverse events: 70.1% vs 69.3%; Dry mouth: 3.1% vs 1.4%	Tiotropium (via Respimat®) significantly improved lung function and HRQoL and reduced exacerbations compared with placebo
Vogelmeier 2011 [39]	7376	• Tiotropium 18 μg QD • Salmeterol 50 μg BID	Time to first exacerbation: 187 days vs 145 days (*p* < 0.001)	Serious adverse events: 14.7% vs 16.5%	Tiotropium significantly reduced exacerbations compared with salmeterol
Wise 2013 [81]	17,135	• Tiotropium 2.5 μg QD • Tiotropium 5 μg QD • Tiotropium 18 μg QD	Deaths: 7.7% vs 7.4% vs 7.7% Proportion of patients with exacerbations: 49.4% vs 47.9% vs 48.9%	Serious adverse events: 33.8% vs 32.4% vs 32.4%	Tiotropium 2.5 μg or 5 μg (via Respimat®) was similar to tiotropium 18 μg (via HandiHaler®) in safety and exacerbation efficacy

*AUC*_*0–12h*_ area under the curve from 0 to 12 h post-dose, *BID* twice a day, *COPD* chronic obstructive pulmonary disease, *DPI* dry-powder inhaler, *FEV*_*1*_ forced expiratory volume in 1 s, *FVC* forced vital capacity, *HRQoL* health-related quality of life, *NA* not available, *pMDI* pressurized metered-dose inhaler, *QD* once daily, *QID* four times a day, *SGRQ* St. George’s Respiratory Questionnaire

^a^Reduction of at least four units in the SGRQ score

*For studies in which the primary endpoint was not specified, results of lung function are included.**Includes the total number of patients in the tiotropium, salmeterol, and placebo groups

Tiotropium 18 μg delivered via HandiHaler®; tiotropium 2.5 μg, 5 μg, and 10 μg delivered via Respimat®; other drugs delivered via a pMDI or a DPI

## Clinical development of tiotropium/olodaterol Respimat®

As mentioned, the clinical development of tiotropium culminated with evidence supporting its use in a fixed-dose combination with olodaterol. The ToVITO® program, which involved more than 16,000 patients and is outside the scope of this review, was designed to evaluate the effect of tiotropium/olodaterol (2.5/5 μg and/or 5/5 μg) Respimat® on various efficacy parameters, as well as its safety. Results of these trials, and others, demonstrated that tiotropium/olodaterol provided a significant, incremental benefit over tiotropium monotherapy in alleviating COPD symptoms such as breathlessness, improving lung function and QoL, and reducing moderate and severe exacerbations (Table 3) [95–102]. In the DYNAGITO® trial, tiotropium/olodaterol reduced the rate of moderate and severe exacerbations by 7% compared with tiotropium alone, which did not meet the predefined significance level of 0.01. However, in a post hoc analysis using multiple covariate models similar to those used in the SPARK and FLAME trials, an 11% reduction in moderate-to-severe exacerbations was observed [102, 103]. Results across trials included in the ToVITO® program showed no significant differences in the frequency of general and serious adverse events between tiotropium/olodaterol Respimat® and mono components [104].

**Table 3:** Tiotropium/olodaterol combination – summary table of evidence

Study	Patients (N)	Treatment arms	Primary endpoint results	Proportion of patients with adverse events	Conclusion of the study
Buhl 2015 [95]	5162	• Tiotropium+olodaterol 2.5/5 μg QD • Tiotropium+olodaterol 5/5 μg QD • Tiotropium 2.5 μg QD • Tiotropium 5 μg QD • Olodaterol 5 μg QD	• FEV_1_ AUC_0–3_ response: o Tiotropium+olodaterol 2.5/5 μg vs olodaterol 5 μg, 0.115 L; vs tiotropium 2.5 μg, 0.111 L; and vs tiotropium 5 μg, 0.097 L (*p* < 0.0001 for all comparisons) o Tiotropium+olodaterol 5/5 μg vs olodaterol 5 μg, 0.128 L and vs tiotropium 5 μg, 0.110 L (*p* < 0.0001 for both) • Trough FEV_1_ response: o Tiotropium+olodaterol 2.5/5 μg: vs olodaterol 5 μg, 0.062 L; vs tiotropium 2.5 μg, 0.045 L; vs tiotropium 5 μg, 0.038 L (*p* < 0.0001 for all comparisons) o Tiotropium+olodaterol 5/5 μg: vs olodaterol 5 μg, 0.085 L; vs tiotropium 5 μg, 0.060 L (*p* < 0.0001 for both comparisons) • SGRQ total score: o Tiotropium+olodaterol 5/5 μg: vs olodaterol 5 μg, − 1.693 (*p* = 0.0022); vs tiotropium 5 μg, − 1.233 (*p* = 0.0252) o Tiotropium+olodaterol 2.5/5 μg vs individual components was not significant for all comparisons	74.7% vs 74.0% vs 73.4% vs 73.3% vs 76.6%	Tiotropium+olodaterol improved lung function and HRQoL compared with monocomponents
Beeh 2015 [96]	259	• Tiotropium+olodaterol 2.5/5 μg QD • Tiotropium+olodaterol 5/5 μg QD • Tiotropium 2.5 μg QD • Tiotropium 5 μg QD • Olodaterol 5 μg QD • Placebo	• FEV_1_ AUC_0–24_ response: o Tiotropium+olodaterol 2.5/5 μg: vs olodaterol 5 μg, 0.111 L; vs tiotropium 2.5 μg, 0.124 L; vs tiotropium 5 μg, 0.107 L; vs placebo, 0.277 L (*p* < 0.001 for all comparisons) o Tiotropium+olodaterol 5/5 μg: vs olodaterol 5 μg, 0.115 L; vs tiotropium 2.5 μg, 0.127 L; vs tiotropium 5 μg, 0.110 L; vs placebo, 0.280 L (*p* < 0.0001 for all comparisons)	36.0% vs 37.4% vs 39.4% vs 44.2% vs 37.7% vs 46.4%	Tiotropium+olodaterol improved lung function over 24 h compared with monocomponents
O’Donnell 2017 [97]	586	• Tiotropium+olodaterol 2.5/5 μg QD • Tiotropium+olodaterol 5/5 μg QD • Tiotropium 5 μg QD • Olodaterol 5 μg QD • Placebo	• Inspiratory capacity: o Tiotropium+olodaterol 2.5/5 μg: vs olodaterol 5 μg, 0.090 L; vs tiotropium 5 μg, 0.092 L; vs placebo, 0.245 L (*p* < 0.0001 for all comparisons) o Tiotropium+olodaterol 5/5 μg: vs olodaterol 5 μg, 0.099 L; vs tiotropium 5 μg, 0.101 L; vs placebo, 0.254 L (*p* < 0.0001 for all comparisons) • Exercise endurance time during constant work-rate cycle ergometry (improvement): o Tiotropium+olodaterol 2.5/5 μg: vs olodaterol 5 μg, 7.3% (*p* < 0.01); vs tiotropium 5 μg, 3.5%; vs placebo, 19.2% (*p* < 0.0001) o Tiotropium+olodaterol 5/5 μg: vs olodaterol 5 μg, 5.6% (*p* < 0.05); vs tiotropium 5 μg, 1.9%; vs placebo, 17.3% (*p* < 0.0001)	36.3% vs 40.0% vs 38.3% vs 40.2% vs 40.8%	Tiotropium+olodaterol improved lung hyperinflation and exercise tolerance compared with monotherapies
Maltais 2018 [98]	404	• Tiotropium+olodaterol 2.5/5 μg QD • Tiotropium+olodaterol 5/5 μg QD • Placebo	• Endurance time during constant work-rate cycle ergometry: o Tiotropium+olodaterol 5/5 μg vs placebo, 14% (*p* = 0.02) o Tiotropium+olodaterol 2.5/5 μg vs placebo, 9% (*p* = 0.14)	54.9% vs 43.9% vs 50.8%	Tiotropium+olodaterol improved endurance time compared with placebo during cycle ergometry
Singh 2015 [99]	1621	• Tiotropium+olodaterol 2.5/5 μg QD • Tiotropium+olodaterol 5/5 μg QD • Tiotropium 5 μg QD • Placebo	• SGRQ total score (difference): o Tiotropium+olodaterol 5/5 μg: vs tiotropium 5 μg, − 2.10 (*p* < 0.01); vs placebo, − 4.67 (*p* < 0.0001) o Tiotropium+olodaterol 2.5/5 μg: vs tiotropium 5 μg, − 1.27; vs placebo, − 3.85 (*p* < 0.0001) • FEV_1_ AUC_0–3_ response: o Both tiotropium+olodaterol 2.5/5 μg and 5/5 μg significantly improved (*p* < 0.0001) FEV_1_ AUC_0–3_ response compared with placebo and tiotropium 5 μg	OTEMTO 1: 42.6% vs 44.8% vs 44.3% vs 51.5% OTEMTO 2: 45.5% vs 43.1% vs 45.8% vs 46.0%	Tiotropium+olodaterol improved lung function and QoL compared with placebo and tiotropium
Beeh 2016 [100]	229	• Tiotropium+olodaterol 2.5/5 μg QD • Tiotropium+olodaterol 5/5 μg QD • Salmeterol/fluticasone 50/500 μg BID • Salmeterol/fluticasone 50/250 μg BID	• FEV_1_ AUC_0–12_ response: o 0.295 L vs 0.317 L vs 0.188 L vs 0.192 L (*p* < 0.0001 for comparisons of tiotropium+olodaterol vs salmeterol/fluticasone)	34.4% vs 33.9% vs 37.0% vs 29.7%	Tiotropium+olodaterol QD provided superior improvement in lung function compared with salmeterol/fluticasone BID
Troosters 2018 [101]	303	• Tiotropium+olodaterol 5/5 μg QD • Tiotropium+olodaterol 5/5 μg QD plus 8 weeks of ExT • Tiotropium 5 μg • Placebo	• Exercise endurance time by shuttle walk test (increase): o Tiotropium+olodaterol 5/5 μg QD vs placebo, 29.2% (*p* = 0.0109) o Tiotropium+olodaterol 5/5 μg QD plus 8 weeks of ExT vs placebo, 45.8% (*p* = 0.0002) o Tiotropium 5 μg vs placebo, 4.1% (*p* = 0.6895)	57.9% vs 64.5% vs 67.1% vs 61.3%	In patients taking part in a self-management behavior-modification program, tiotropium+olodaterol improved exercise endurance time compared with placebo
Calverley 2018 [102]	7880	• Tiotropium+olodaterol 5/5 μg QD • Tiotropium 5 μg QD	• Rate of moderate and severe COPD exacerbations: o 0.90 vs 0.97 (rate ratio, 0.93; *p* = 0.0498)	74% vs 75%	Tiotropium+olodaterol reduced exacerbation rate compared with tiotropium, but not to a significant extent

*AUC*_*0–24*_ area under the curve from 0 to 24 h post-dose, *AUC*_*0–12*_ area under the curve from 0 to 12 h post-dose, *AUC*_*0–3*_ area under the curve from 0 to 3 h post-dose, *BID* twice a day, *COPD* chronic obstructive pulmonary disease, *ExT exercise training, FEV*_*1*_ forced expiratory volume in 1 s, *HRQoL* health-related quality of life, *QD* once daily, *QoL* quality of life, *SGRQ* St. George’s Respiratory Questionnaire

Tiotropium will continue to be the first-line treatment for newly diagnosed COPD, in particular in mild to moderate cases and infrequent exacerbators. We hope that early diagnosis will be more frequent in the future and these newly diagnosed cases, probably with milder disease, might be optimal candidates for treatment with tiotropium/olodaterol if new studies demonstrate that optimal early bronchodilation results in improved long-term outcomes.

## Conclusions

Treatment goals for COPD include reduction in symptoms and future risk of exacerbations [1, 2, 105]. Per GOLD recommendations, LAMA monotherapy is recommended as initial treatment for GOLD groups B, C, and D. LABA/LAMA combination therapy may be considered in group B patients with severe breathlessness and is an initial treatment option for group D patients who are highly symptomatic [2]. No other LAMA has been proven superior to tiotropium, making it an optimal LAMA as monotherapy and as the backbone LAMA in LABA/LAMA combination therapy. In most comparative studies of tiotropium and placebo, ipratropium, or salmeterol, tiotropium provided significant beneficial effects on lung function, including improvements in FEV_1_ and FVC [21–36]. Tiotropium also significantly improved exacerbation-related outcomes such as reduction in the number of exacerbations/PY, reduction in the proportion of patients experiencing ≥1 exacerbation and exacerbation-related hospitalizations, increase in the time to first exacerbation, and reduction in HCRU compared with placebo and salmeterol [22–24, 26, 28, 34, 36–39]. In addition, tiotropium treatment improved HRQoL and significantly reduced dyspnea, need for “as-needed” SABA use, and lung hyperinflation, resulting in improvement in exertional dyspnea and exercise endurance [21–23, 26, 28, 29, 32, 34–36, 45, 46]. The long-term efficacy of tiotropium was demonstrated in the UPLIFT trial. Finally, tiotropium was comparable to ICS/LABA (fluticasone/salmeterol) in improving lung function and reducing exacerbations [66, 67] and had a greater effect on exacerbation rates than LABAs [39, 73]. Because long-term use of ICS is associated with systemic and local side effects, tiotropium is a suitable alternative to ICS/LABA combinations. Overall, tiotropium is safe and efficacious as a long-term, QD LAMA for the maintenance treatment of COPD and for reducing COPD exacerbations or for the maintenance treatment of COPD as part of long-term, QD, fixed-dose LAMA/LABA (tiotropium/olodaterol) [6, 7, 9].

## Supplementary information

**Additional file 1.** Tiotropium in chronic obstructive pulmonary disease – a review of clinical development. An infographic that provides a summary of the clinical trial results of tiotropium in chronic obstructive pulmonary disease

## Data Availability

Not applicable.

## Abbreviations

AUC_0–24_: area under the curve for the 24-h period post-morning dose
BID: twice daily
CI: confidence interval
COPD: chronic obstructive pulmonary disease
DPI: dry-powder inhaler
EU: European Union
FEV_1_: forced expiratory volume in 1 s
FVC: forced vital capacity
GLOW: glycopyrronium bromide in COPD airways
GOLD: Global Initiative for Chronic Obstructive Lung Disease
HCRU: health care resource utilization
HR: hazard ratio
HRQoL: health-related quality of life
IC: inspiratory capacity
ICS: inhaled corticosteroid
INHANCE: INdacaterol to Help Achieve New COPD treatment Excellence
INSPIRE: Investigating New Standards for Prophylaxis in Reducing Exacerbations
INTENSITY: INdacaterol Towards Establishment of cliNical SuperiorITY study
INTIME: INdacaterol & TIotropium: Measuring Efficacy
INVIGORATE: Indacaterol: Providing Opportunity to Re-engage Patients with Life
LABA: long-acting β_2_-agonist
LAMA: long-acting muscarinic antagonist
MISTRAL: Mesure de l’Influence de Spiriva® sur les Troubles Respiratoires Aigus à Long terme
OR: odds ratio
PEFR: peak expiratory flow rate
pMDI: pressurized metered-dose inhaler
POET-COPD: Prevention Of Exacerbations with Tiotropium in COPD
PY: patient-year
QD: once daily
QID: four times a day
RCT: randomized controlled trial
RR: rate ratio
SABA: short-acting β_2_-agonist
SAMA: short-acting muscarinic antagonist
SGRQ: St. George’s Respiratory Questionnaire
SMI: soft mist inhaler
TDI: transition dyspnea index
TIOSPIR: Tiotropium Safety and Performance In Respimat
TIPHON: Tiotropium: Influence sur la Perception de l’amélioration des activites Habituelles Objectivée par une echelle Numerique
UPLIFT: Understanding Potential Long-Term Impacts on Function with Tiotropium
US: United States
