# Supplementary material for: Tiotropium in chronic obstructive pulmonary disease – a review of clinical development
Source: Respir Res. 2020 Jul 29;21:199. doi: 10.1186/s12931-020-01407-y (PMC7389564; doi:10.1186/s12931-020-01407-y)
Supplement: Supplementary file 1 — Additional file 1. Tiotropium in chronic obstructive pulmonary disease – a review of clinical development. An infographic that provides a summary of the clinical trial results of tiotropium in chronic obstructive pulmonary disease [file 12931_2020_1407_MOESM1_ESM.pdf]

Clinical Development of **Tiotropium HandiHaler®** in **COPD**

Category	Outcome	Tiotropium HandiHaler® dose-response study (vs placebo)[1]	Tiotropium HandiHaler® 18 µg QD 3-month RCT (vs placebo)[2]	Tiotropium HandiHaler® 18 µg QD pivotal 6-month/1-year RCTs (vs ipratropium, salmeterol, or placebo)[3-9]	Tiotropium HandiHaler® 18 µg QD additional RCTs (vs placebo)[10-12]	Tiotropium HandiHaler® 18 µg QD RCTs (vs ICS/LABA, SAMA/SABA, LABA, or other LAMAs)[13-22]
Lung parameters	Lung function	↑	↑	↑	↑	≈ vs ICS/LABA, LABA, other LAMAs<br>↑ vs SAMA/SABA
	Lung hyperinflation				↓	
	Inspiratory capacity			↑ vs placebo	↑	≈ vs LAMA
Symptoms & PROs	Dyspnea		↓	↓	↓ exertional dyspnea	≈ vs LABA or other LAMAs
	Wheezing		↓			
	Endurance				↑	
	Rescue medication use		↓	↓ vs placebo & ipratropium		≈ vs SAMA/SABA
	HRQoL			↑	↑	≈ vs LABA or other LAMAs
Exacerbations & hospitalizations	Exacerbations			↓ exacerbations<br>↑ time to 1st exacerbation	↓	≈ vs ICS/LABA & other LAMAs<br>↓ risk vs SAMA/SABA<br>↓ annualized rate vs LABA
	Exacerbation- or COPD-related hospitalization			↑ time to 1st exacerbation-related hospitalization vs placebo & ipratropium<br>↓ exacerbation-related hospitalization vs placebo		↓ COPD-related hospitalization vs SAMA/SABA
Safety	Safety	≈	≈ overall AEs<br>↑ incidences of dry mouth	≈ overall AEs<br>↑ incidences of dry mouth	≈ overall AEs<br>↑ incidences of dry mouth & pharyngeal discomfort	≈ overall AEs<br>↓ risk of pneumonia vs ICS/LABA

↑ improved; ↓ reduced; ≈ comparable.

AE, adverse event; COPD, chronic obstructive pulmonary disease; HRQoL, health-related quality of life; ICS, inhaled corticosteroid; LABA, long-acting β_2-agonist; LAMA, long-acting muscarinic antagonist; PRO, patient-reported outcome; QD, once daily; RCT, randomized controlled trial; SABA, short-acting β_2-agonist; SAMA, short-acting muscarinic antagonist.

1. Littner MR, et al. *Am J Respir Crit Care Med*. 2000;161:1136-1142; **2.** Casaburi R, et al. *Chest*. 2000;118:1294-1302; **3.** Donohue JF, et al. *Chest*. 2002;122:47-55; **4.** Brusasco V, et al. *Thorax*. 2003;58:399-404; **5.** Vincken W, et al. *Eur Respir J*. 2002;19:209-216; **6.** Casaburi R, et al. *Eur Respir J*. 2002;19:217-224; **7.** Niewoehner DE, et al. *Ann Intern Med*. 2005;143:317-326; **8.** Dusser D, et al. *Eur Respir J*. 2006;27:547-555; **9.** Vogelmeier C, et al. *N Engl J Med*. 2011;364:1093-1103; **10.** O'Donnell DE, et al. *Eur Respir J*. 2004;23:832-840; **11.** Zhou Y, et al. *N Engl J Med*. 2017;377:923-935; **12.** Tashkin DP, et al. *N Engl J Med*. 2008;359:1543-1554; **13.** Bateman ED, et al. *Pulm Pharmacol Ther*. 2008;21:20-25; **14.** Wedzicha JA, et al. *Am J Respir Crit Care Med*. 2008;177:19-26; **15.** Niewoehner DE, et al. *Pulm Pharmacol Ther*. 2009;22:587-592; **16.** Griffin J, et al. *Prim Care Respir J*. 2008;17:104-110; **17.** Vogelmeier C, et al. *Respir Res*. 2010;11:135; **18.** Buhl R, et al. *Eur Respir J*. 2011;38:797-803; **19.** Donohue JF, et al. *Am J Respir Crit Care Med*. 2010;182:155-162; **20.** Decramer ML, et al. *Lancet Respir Med*. 2013;1:524-533; **21.** Beier J, et al. *COPD*. 2013;10:511-522; **22.** Chapman KR, et al. *BMC Pulm Med*. 2014;14:4

Clinical Development of **Tiotropium Respimat®[23]** in COPD

Clinical Development of **Tiotropium + Olodaterol Respimat®** in COPD

Category	Parameter	Tiotropium Respimat® dose-response studies (vs tiotropium HandiHaler® or placebo)[24,25]	Tiotropium Respimat® 5 µg & 10 µg QD pivotal RCTs (vs ipratropium or placebo)[26-28]	Tiotropium Respimat® 2.5 µg & 5 µg QD landmark RCT & post hoc analyses (vs tiotropium HandiHaler® 18 µg QD)[29-31]	Tiotropium + olodaterol Respimat® 2.5/5 µg or 5/5 µg RCTs (vs tiotropium, olodaterol, ICS/LABA, or placebo)[32-44]
Lung parameters	Lung function	↑ vs placebo	↑		↑
	Lung hyperinflation				↓
	Inspiratory capacity				↑ vs tiotropium and olodaterol
	Bronchodilation			≈	
Symptoms & PROs	Dyspnea		↓ vs placebo		↓
	Endurance				↑
	Rescue medication use	≈ vs tiotropium HandiHaler®<br>↓ vs placebo	↓		
	HRQoL		↑ vs placebo		↑
Exacerbations & hospitalizations	Exacerbations		↓ exacerbation rate<br>↓ patients with ≥1 exacerbation<br>↑ time to 1st exacerbation vs placebo	Nonsuperior risk of 1st exacerbation<br>↑ risk of exacerbation with ICS use	↓ vs tiotropium
Safety parameters	Safety	≈ overall AEs	≈ overall AEs<br>↑ incidences of dry mouth	≈	≈ overall AEs
	Risk of death			Noninferior	

↑ improved; ↓ reduced; ≈ comparable.

23. Dalby RN, et al. *Med Devices*. 2011;4:145-155; **24.** van Noord JA, et al. *Respir Med*. 2009;103:22-29; **25.** Caillaud D, et al. *Int J Chron Obstruct Pulmon Dis*. 2007;2:559-565; **26.** Voshaar T, et al. *Respir Med*. 2008;102:32-41; **27.** Bateman ED, et al. *Int J Chron Obstruct Pulmon Dis*. 2010;5:197-208; **28.** Bateman ED, et al. *Respir Med*. 2010;104:1460-1472; **29.** Wise RA, et al. *N Engl J Med*. 2013;369:1491-1501; **30.** Anzueto A, et al. *Respir Res*. 2015;16:107; **31.** Calverley PM, et al. *Int J Chron Obstruct Pulmon Dis*. 2017;12:3391-3405; **32.** Ferguson F, et al. *Adv Ther.* 2015;32:523-526; **33.** Buhl R, et al. *Eur Respir J*. 2015;45:969-979; **34.** Beeh KM, et al. *Pulm Pharmacol Ther*. 2015;32:53-59; **35.** O'Donnell DE, et al. *Eur Respir J*. 2017;49:1601348; **36.** Maltais F, et al. *Ther Adv Respir Dis*. 2018;12:1753465818755091; **37.** Singh D, et al. *Respir Res*. 2016;17:73; **38.** Singh D, et al. *Respir Med*. 2015;109:1312-1319; **39.** Beeh KM, et al. *Int J Chron Obstruct Pulmon Dis*. 2016;11:193-205; **40.** Troosters T, et al. *Am J Respir Crit Care Med*. 2018;198:1021-1032; **41.** Bourbeau J, et al. *BMJ Open.* 2016;6:e010109; **42.** Troosters T, et al. *BMJ Open.* 2016;6:e010106; **43.** Calverley PMA, et al. *Lancet Respir Med*. 2018;6:337-344; **44.** Maltais F, et al. *Am J Respir Crit Care Med*. 2018;197:A2373.

Disclosure: The authors meet the criteria for authorship as recommended by the International Committee of Medical Journal Editors (ICMJE). The authors received no direct compensation related to the development of this infographic. Writing, editorial support, and formatting assistance was provided by Suchita Nath-Sain, PhD, Saurabh Gagangras, PhD, Praveen Kaul, PhD, and Maribeth Bogush, PhD, of Cactus Communications, which was contracted and funded by Boehringer Ingelheim Pharmaceuticals, Inc. (BIPI). BIPI was given the opportunity to review the infographic for medical and scientific accuracy, as well as intellectual property considerations.
